# Central and Extrapontine Myelinolysis

**DOI:** 10.5334/jbr-btr.1145

**Published:** 2016-06-30

**Authors:** Charlemagne Noukoua

**Affiliations:** 1 André Renard hospital liege, BE

**Keywords:** Central and extrapontine myelinolysis, Hyponatremia

A 48-year-old man with a background of alcohol abuse was found at home by the emergency services with impaired psychomotor status. According to his relatives, he had recently attempted to reduce his consumption of alcohol. However, a dozen empty bottles of beer were found around him. On admission, he complained of headache and presented with confusion and walking difficulties. He had a scalp injury that was consistent with a possible fall.

Except for a slight tremor, the neurological examination was normal. Blood testing revealed low levels of sodium (112 mmol/L; normal values (nv) 135–148), potassium (3.19 mmol/L; nv 3.5–5.5), and chloride (63 mmol/L; nv 98–110). Computed tomography (CT) failed to reveal any sign of brain trauma. The patient was then admitted to the neurological department with the diagnosis of alcohol-related hyponatremia and hypokaliema.

During the following days, the treatment resulted in normalization of the blood ions. Ten days later, however, the patient exhibited increasing neurological disorders, including dysarthria, tremors, swallowing difficulty, impaired reflexes, quadriplegia, and loss of consciousness that progressed to locked-in-syndrome status fourteen days after admission. As the electroencephalogram showed findings consistent with severe encephalopathy at this time, the patient underwent brain magnetic resonance imaging (MRI) (Figure 1). It revealed confluent areas of edema (arrows), displaying high signal intensity on T2-weighted fluid-attenuated inversion recovery (FLAIR) (Figures B and D) and low signal intensity on T1-weighted (Figures A and C) images in the brainstem and symmetrically in the basal ganglia and the brain’s white matter, consistent with the diagnosis of central pontine myelinolysis (CPM) and extrapontine myelinolysis (EPM).

**Figure 1:**
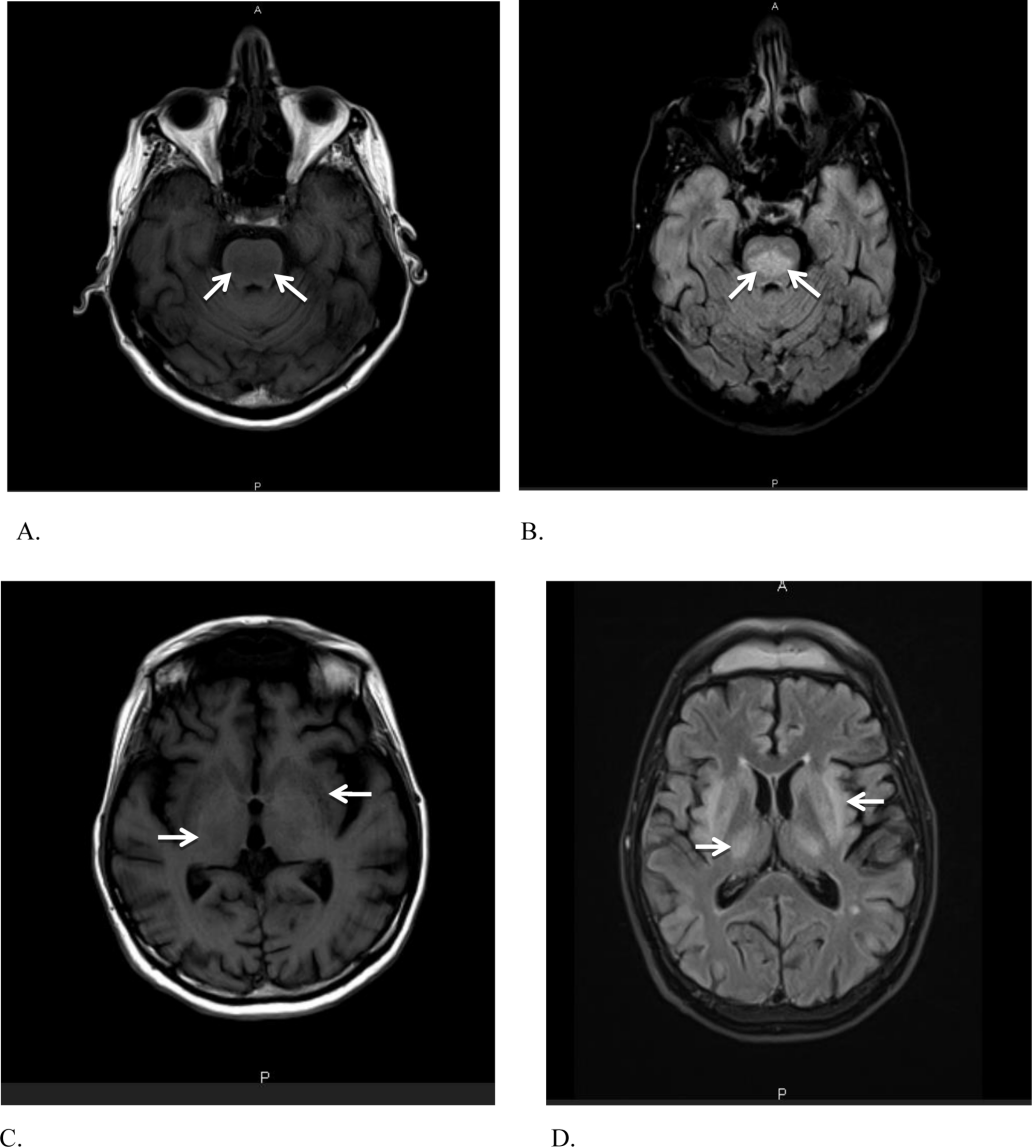


The treatment was adapted by diminution of sodium intake, and the symptoms and the neurological status of the patient improved dramatically, returning to usual state 72 hours later.

## Discussion

Central myelinolysis was first described in 1959 by Adams as demyelinating lesions occurring in the central pons in alcoholic and undernourished patients. Though CPM can occur with or without EPM, both share the same pathological pathways but differ in their clinical manifestations. Their etiology and incidence are not documented in a detailed manner, but they have been associated with rapid correction of hyponatremia. The risk factors include chronic hyponatremia, alcohol withdrawal, malnutrition, liver failure, and immune-compromised system. It is believed that chronic hyponatremia is associated with loss of electrolytes (sodium, potassium, chloride) and water from brain cells. Aggressive correction of hyponatremia alters the balance of the osmotic forces between the interstitial space, brain cells, and blood space, facilitating a net water movement towards the interstitium and the cells. This leads to the destruction of the myelin sheath and induces brain and brainstem swelling without inflammation. The preferred location of osmotic myelinolysis in the pons is related to the susceptibility of oligodendrocytes —that make up most of the transverse myelin sheath compostition—to dehydratation and volume changes.

The clinical presentation of EPM and CPM correlate with the size and the location of the lesions. In the early stage of the disease, transitory symptoms such as confusion and agitation are associated with encephalopathy-induced hyponatremia. This situation may improve slightly with the correction of the natremia, but the later stages begin when this correction induces myelinolysis and manifests with progressive neurological signs, as in our patient. CT has low sensitivity to show lesions induced by acute myelinolysis because the pontine area imaging is often degraded by artifacts. Nevertheless, the lesions generally appear lately with low density in the brain and the pons. MRI is the standard of reference for all lesions, especially early lesions which appear symmetrically in the basal ganglia, in the subcortical white matter, and/or in the central pons. Typically, the lesions shorten the T2 and show neither change on diffusion nor enhancement after contrast administration. Partial enhancement of the lesions is, however, possible in the early stage [1].

Left untreated, the prognosis of EPM/CPM is poor and death usually occurs during the first two weeks, whereas appropriate fluid and ionic management improves the clinical outcome, stressing the key importance of MRI findings awareness for early diagnosis and improved patient outcomes. Lastly, it should be acknowledged that MRI abnormalities neither correlate with the severity of the symptoms nor follow the clinical course after appropriate management. Indeed, while the lesion’s edema disappears with time, gliosis may cause definitive T2-weighted shortening.
